# Metastatic renal cell carcinoma to the jaws: report of cases

**DOI:** 10.1186/1477-7819-12-204

**Published:** 2014-07-11

**Authors:** Louqiang Zhang, Hongbin Yang, Xuebin Zhang

**Affiliations:** 1 Department of Stomatology, Tianjin Medical University General Hospital, No154, Anshan Road, Heping District, Tianjin 300052, People’s Republic of China; 2 Department of Pathology, Tianjin Huanhu Hospital, No122, Qi Xiang Tai Road, Hexi District, Tianjin 300060, People’s Republic of China

**Keywords:** Mandible, Maxilla, Metastasis, RCC, Renal cell carcinoma

## Abstract

Renal cell carcinoma (RCC) is one of the most frequent urological malignancies in adults. RCC often metastasizes to other organs, but rarely to the oromaxillofacial region. Metastatic tumors to the jaws are also unusual. In this report, we present two cases of RCC metastasis to the jaws. Metastatic RCC is resistant to radiotherapy and chemotherapy, so surgery is the primary therapeutic choice. This report describes the diagnostic procedures utilized and the therapeutic process in the two cases. The differential diagnosis and treatment methods are discussed.

## Background

Jawbone metastases are rare; they account for less than 1% of oral and maxillofacial malignancies. The most common malignant primary lesions that metastasize to the jawbones arise from the lung, kidney, prostate and rectum in men and from the breast, kidney, uterus and thyroid in women. The mandibular body and ramus are common sites of metastases, and maxillary metastases are uncommon.

Adult renal cell carcinoma (RCC) is a neoplasm of the urinary system that accounts for 3% of adult malignancies and 90% to 95% of kidney tumors [1]. RCC is most common in men ages 50 to 60 years old [2]. Only 10% of the patients exhibit the classic Grawitz triad (flank pain, palpable mass and hematuria) [3-5]. Thirty percent of patients have a distant metastasis [6,7], most commonly to the lung, followed by bone, liver, brain and regional lymph nodes, but seldom to the mandible or the maxilla. In this report, we describe the cases of two patients,one with maxillary and the other with mandibular metastasis of RCC.

## Case presentations

### Patient 1

**Figure 1:**
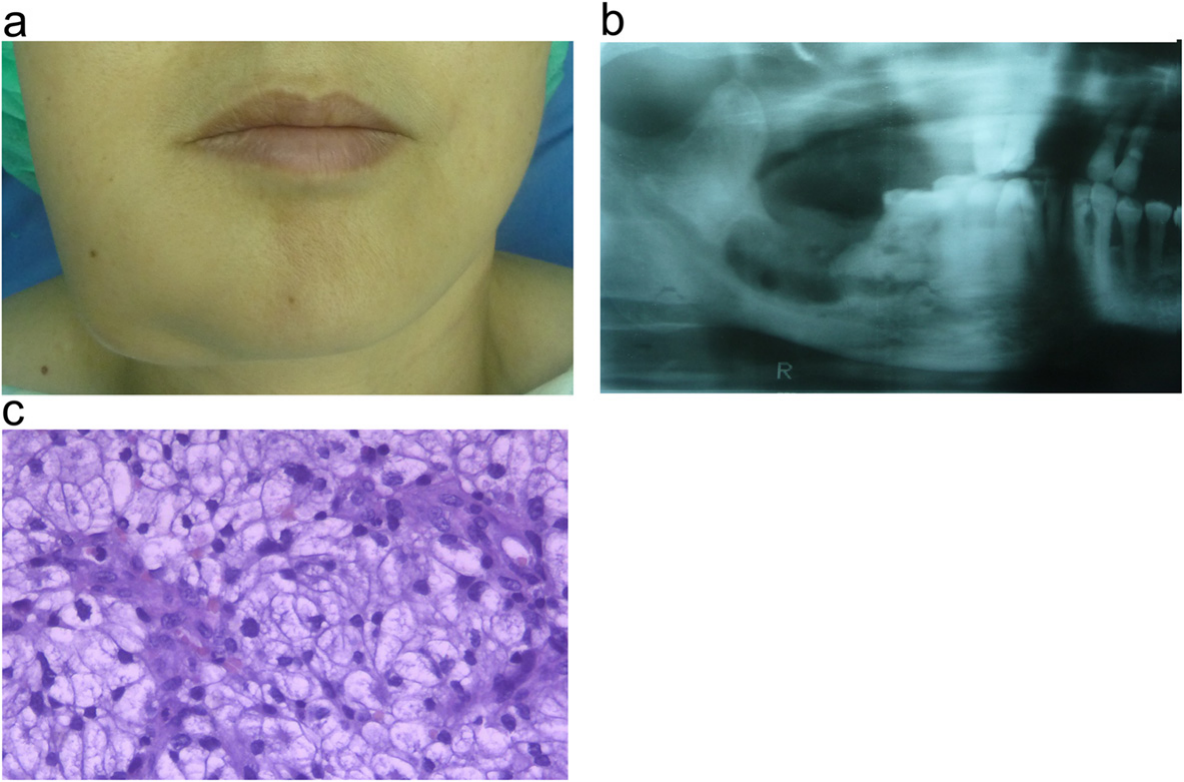
**Case 1: renal cell carcinoma metastasis to the mandible. (a)** Photograph showsswelling of the right side of the patient’s jaw due to the mandibular tumor. **(b)** Radiograph showsosteolytic damage in the right mandibular body. **(c)** Hematoxylin and eosin–stained tumor section.

A 45-year-old man presented the department of stomatology, Tianjin Medical University General Hospital with swelling of the right-side mandibular body associated with pain during chewing of 1 month’s duration. A mass in the right mandibular body measuring approximately 4.0 × 3.0 × 2.5 cm with a clear border was visible. The buccal bony plate of the mandible had no table tennis sense, but the lingual bony plate was destroyed and the mass bulged out. The texture of the mass was soft, and there was right lower lip paresthesia. An X-ray examination revealed an intraosseous mass with lytic damage of the right mandibular body (Figure 1). The patient’s right kidney had been removed 2 years prior for RCC, and he had been receiving 200 million units of interleukin2 every week since the operation to the presentation time. When the patient was admitted, metastatic cancer was not initially considered;a primary tumor was favored. Metastatic RCC to the mandible was confirmed following biopsy. Under the microscope, we saw that the tumor cells were largeand cuboidal, with some columnar, and that most of the cancer cells in the cytoplasm were transparent. Cellular atypia and mitosis were visible. The interstitial tissues were rich in capillaries. Immunohistochemical staining showed that the tumor was CAM5.2+, renal cell carcinoma marker–positive (RCC-Ma+), CD10+, vimentin-positive (VIM+) and Ki67+ .

### Patient 2

**Figure 2:**
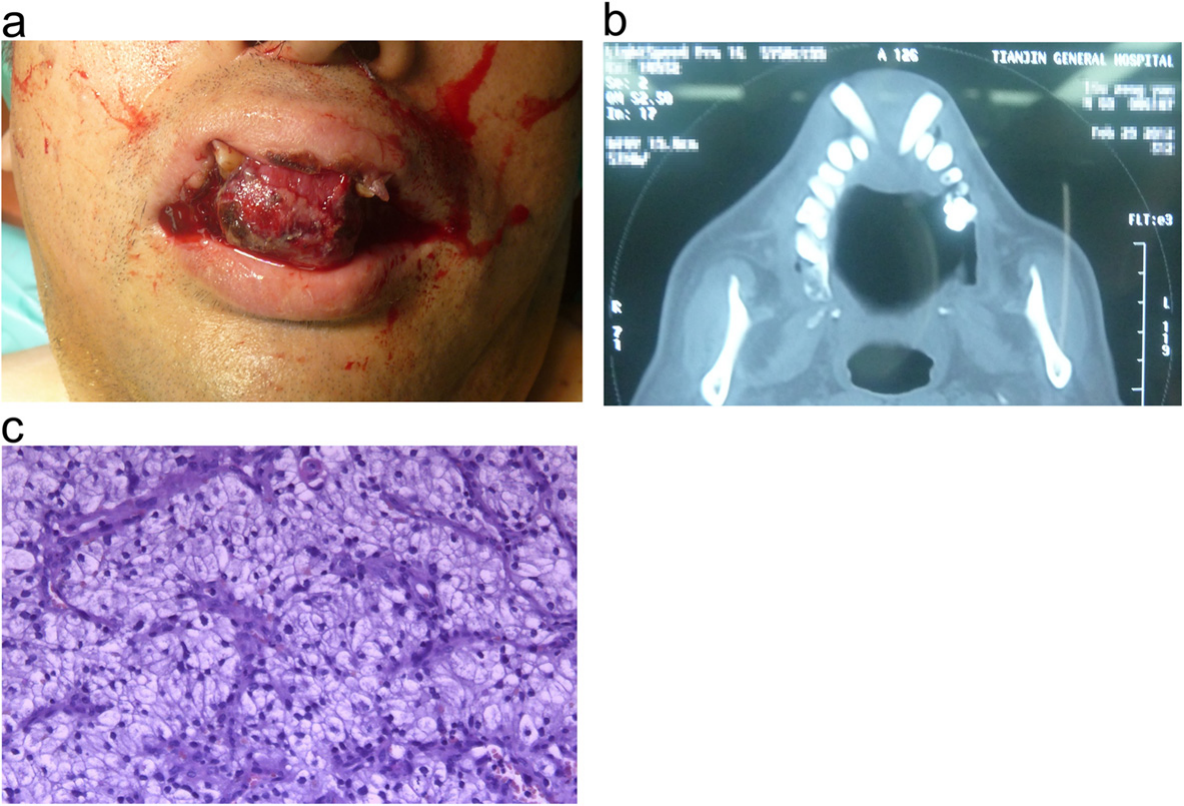
**Case 2: renal cell carcinoma metastasis to the maxilla. (a)** Photograph shows the patient’s maxillary tumor. **(b)** Computed tomography scan shows the osteolytic damage in the maxilla. **(c)** Hematoxylin and eosin–stained tumor section.

A 60-year-old man with a 4-year history of RCC of the left kidney presented the department of stomatology, Tianjin Medical University General Hospital with a slowly growing mass in the maxilla of 20 days’ duration. The mass was located at the palatal side of the anterior teeth and measured approximately 6.0 × 4.0 × 3.0 cm with an irregular outline. The surface was ulcerated, with an overlying pseudomembranous layer and hemorrhage. A computed tomography scan showed lytic destruction of the alveolar bone (Figure 2). The patient had been noted to have a lung metastasis 2 months earlier. In contrast to case 1, a diagnosis of metastatic RCC was readily made on the basis of the presence of existing systemic metastases. The tumor was resected later. Expansion of the blood vessels,along with bleeding and a hemosiderin deposit,were seen under the microscope. Cellular atypia and mitosis were visible, the tumor cells were largeand the cytoplasm was transparent. Immunohistochemical staining showed that the tumor was CAM5.2+, RCC-Ma+, CD10+, VIM + and Ki67+ .

### Differential diagnosis

Malignant tumors of the jaw can cause lip paresthesia, numbness, jaw swelling, pain and tooth mobility. These symptoms are often persistent and progressive. X-rays typically show irregular osteolytic destruction.

Oral and maxillofacial surgeons should distinguish jaw metastases of RCC from central mandibular carcinoma, squamous cell cancer, carcinoma arising from salivary glands (such as mucoepidermoid, myoepithelial, epithelial–myoepithelial and acinar cell carcinoma) and benign gum diseases (such as epulis). Metastases also should be differentiated from other primary clear-cell tumors, which are odontogenic or gland-derived. Consequently, the identification of mandibular metastatic RCC is relatively difficult and requires a high index of suspicion by clinicians. A previous history of primary RCC is crucial to making a definitive diagnosis. If the jaw tumor has a rich blood supply and the patienthas a history of RCC, then metastatic RCC should be considered in the differential diagnosis.

### Treatment

A biopsy was performed for patient 1to confirm the diagnosis. The patient had severe bleeding during surgery. Hemorrhage continued despite the use of hemostatic agents, suture hemostasis and adrenaline package oppression. The bleeding was controlled by tightly packing the tumor bone cavity with gauze. The patient required a 1,000-ml whole-blood transfusion. He refused follow-up segmental resection of the mandible after receiving his diagnosis. He was referred to the oncology department for palliative care.

Patient 2 underwent complete curettage of the metastatic tumor with additional resection of the surrounding alveolar process. Although the patient had severe hemorrhage during surgery, the bleeding was relatively easy to control because of the relatively superficial location of the metastasis. The patient’s hemoglobin remained within the normal range following surgery, and he thus did not require transfusion. Follow-up interferon therapy was administered.

RCC has a propensity to metastasize to another organs, including bilateral adrenal glands [8], skin [9,10], pancreas and spleen [11], gastric or duodenal systems [12], skeleton [13], pancreas [14] and bladder [15,16]. However, metastases from RCC rarely affect the head and neck region [17].

Metastatic RCC is resistant to radiotherapy and chemotherapy, so supportive care and surgery remain the principal treatments of metastatic disease [6]. If the primary tumor has been or can be excised, then metastatic lesions in the jaws should be resected. The risk of hemorrhage is high in these cases, and preoperative vascular embolization to reduce bleeding is recommended [18-20].

The mandibular metastasis case reported in this article was not considered to be a metastatic cancer at the time of patient admission, because the tumor clinically mimicked other localized hyperplasic lesions of the jaw. Patient 1 underwent biopsy and surgery, with resultant intraoperative bleeding similar to the central hemangioma of the mandible. The tumor in the transferred maxillarycarcinoma patient was initially considered to have a metastatic RCC focus. The location of the tumor was relatively superficial and the operative field broad, so intraoperative bleeding was easy to control.

## Conclusions

Metastases from internal neoplasms should be considered among other differential diagnoses in the evaluation of jaw tumors. Especially for patients with a history of RCC, the possibility of metastatic disease should not be ignored. Because jaw metastases of RCC have an extensive supplementary blood supply, appropriate preparation to prevent the risk of bleeding is required prior to biopsy and definitive surgery.

## Consent

Written informed consent was obtained from the patients for publication of this case report and any accompanying images. A copy of the written consent is available for review by the Editor-in-Chief of this journal.

## Competing interests

The authors declare that they have no competing interests.

## Authors’ contributions

LQZ drafted the manuscript,searched the literatureand was involved in the treatment of the patients. HBY prepared the photographs and was involved in the treatment of the patients. XBZ executed the immunohistochemistry that contributed tothe definitive diagnosis and also searched the literature. All authors read and approved the final manuscript.
